# d-Amino acid oxidase and serine racemase in human brain: normal distribution and altered expression in schizophrenia

**DOI:** 10.1111/j.1460-9568.2007.05769.x

**Published:** 2007-09

**Authors:** Louise Verrall, Mary Walker, Nancy Rawlings, Isabel Benzel, James N. C. Kew, Paul J. Harrison, Philip W. J. Burnet

**Affiliations:** 1 Department of Psychiatry, Warneford Hospital, Warneford Lane, University of Oxford Oxford, UK; 2 Psychiatry DTG, GlaxoSmithKline Harlow, Essex, UK

**Keywords:** DAAO, DAO, d-serine, mRNA, SRR

## Abstract

The *N*-methyl-d-aspartate receptor co-agonist d-serine is synthesized by serine racemase and degraded by d-amino acid oxidase. Both d-serine and its metabolizing enzymes are implicated in *N*-methyl-d-aspartate receptor hypofunction thought to occur in schizophrenia. We studied d-amino acid oxidase and serine racemase immunohistochemically in several brain regions and compared their immunoreactivity and their mRNA levels in the cerebellum and dorsolateral prefrontal cortex in schizophrenia. d-Amino acid oxidase immunoreactivity was abundant in glia, especially Bergmann glia, of the cerebellum, whereas in prefrontal cortex, hippocampus and substantia nigra, it was predominantly neuronal. Serine racemase was principally glial in all regions examined and demonstrated prominent white matter staining. In schizophrenia, d-amino acid oxidase mRNA was increased in the cerebellum, and as a trend for protein. Serine racemase was increased in schizophrenia in the dorsolateral prefrontal cortex but not in cerebellum, while serine racemase mRNA was unchanged in both regions. Administration of haloperidol to rats did not significantly affect serine racemase or d-amino acid oxidase levels. These findings establish the major cell types wherein serine racemase and d-amino acid oxidase are expressed in human brain and provide some support for aberrant d-serine metabolism in schizophrenia. However, they raise further questions as to the roles of d-amino acid oxidase and serine racemase in both physiological and pathophysiological processes in the brain.

## Introduction

The *N*-methyl-d-aspartate receptor (NMDAR) is activated by binding of the neurotransmitter glutamate and an obligatory co-agonist, glycine or d-serine. d-Serine is proposed as the predominant co-agonist, at least in the telencephalon (Schell *et al.*, 1995, 1997), consistent with evidence that it modulates cortical and hippocampal NMDAR activity (Thiels *et al*., 1992; Mothet *et al*., 2000; Ito & Hicks, 2001; Yang *et al*., 2003; Shleper *et al*., 2005).

NMDAR hypofunction is considered central to schizophrenia pathophysiology (Olney & Farber, 1995; Coyle, 1996; Moghaddam, 2003), and perhaps to its genetic predisposition (Harrison & Owen, 2003; Harrison & Weinberger, 2005). Impaired d-serine function in patients could contribute to NMDAR hypofunction, as central and peripheral d-serine levels are reduced in schizophrenic patients (Hashimoto *et al*., 2003, 2005; Yamada *et al*., 2005). Moreover, co-administration of d-serine or related compounds can ameliorate some symptoms of the disorder (Tsai *et al*., 1998; Heresco-Levy *et al*., 2005).

Altered d-serine in schizophrenia may be explained, in part, by involvement of the d-serine catabolic enzyme d-amino acid oxidase (DAO, also abbreviated to DAAO). DAO has been associated with susceptibility to schizophrenia (Chumakov *et al*., 2002; Liu *et al*., 2004; Schumacher *et al*., 2004; Corvin *et al*., 2007; Wood *et al*., 2007; but see Yamada *et al*., 2005; Liu *et al*., 2006; Vilella *et al*., 2007) and in addition, its putative activator, G72 (DAOA), also shows association with the disorder (Chumakov *et al*., 2002; Detera-Wadleigh & McMahon, 2006; Corvin *et al*., 2007; Li & He, 2007; Yue *et al*., 2007; but also see Liu *et al*., 2006; Wood *et al*., 2007). DAO has been shown to reduce NMDAR-mediated neurotransmission (Mothet *et al*., 2000) and thus increased DAO in schizophrenia might contribute, via d-serine decrements, to NMDAR dysfunction. Recent studies provide some support for this notion (Kapoor *et al*., 2006; Bendikov *et al*., 2007).

Serine racemase (SRR) synthesizes d-serine from l-serine (Wolosker *et al*., 1999a, b), and thus alterations in SRR activity could enhance or counteract the postulated role of DAO in schizophrenia. At present, there is weak evidence that SRR itself may be a susceptibility gene (Goltsov *et al*., 2006; Morita *et al*., 2007, but see Yamada *et al*., 2005; Strohmaier *et al*., 2007), and evidence that its expression may be altered in the disorder (Steffek *et al*., 2006; Bendikov *et al*., 2007).

The present study had two objectives. First, to characterize the regional and cellular expression of DAO and SRR in human brain. For these studies, we used immunohistochemistry and *in situ* hybridization and focused on areas of relevance to schizophrenia and where DAO is reputed to be abundant or important, namely the dorsolateral prefrontal cortex (DPFC), hippocampus, cerebellum and substantia nigra (Katagiri *et al*., 1991; Horiike *et al*., 1994; Schell *et al*., 1995; Brannan *et al*., 1996; Moses *et al*., 1996; Wang & Zhu, 2003; Kapoor *et al*., 2006; Wu *et al*., 2006; Kawazoe *et al*., 2007). Our second objective was to extend the current data regarding altered expression of DAO and SRR in schizophrenia. For these studies we used real-time PCR and Western blotting as more robust methods to determine mRNA and protein abundance, respectively, and focused on the DPFC and cerebellum, given the reported preferential localizations of SRR and DAO in these regions.

## Materials and methods

### Subjects studied

Brain tissue was collected at autopsy with consent at two centres, Oxford and London. Demographic details of the subjects are shown in Table 1. Schizophrenia was diagnosed from case notes according to DSM-III-R (American Psychiatric Association, 1987) criteria. Most cases were initially of paranoid or disorganized subtype. All patients had received conventional antipsychotics. Control individuals had no history of psychiatric of neurological illness. Neuropathological assessment of all brains did not identify any neurodegenerative abnormalities other than minor age-related changes. For further details see Eastwood *et al*. (2001b). The research was approved by the Oxfordshire Psychiatric Research Ethics Committee.

**Table 1:** Demographic details of schizophrenia patients and control individuals

	Oxford series		London series		Combined series	
			
	Controls	Patients	Controls	Patients	Controls	Patients
Number	12	12	10	9	22	21
Sex (M:F)	6 : 6	7 : 5	6 : 4	5 : 4	12 : 10	12 : 9
Age (years)	65 ± 4.3	53 ± 5.1	63 ± 6.7	62 ± 6.4	64 ± 3.7	57 ± 4.0
(Range)	(25–79)	(19–71)	(26–85)	(32–87)	(25–85)	(19–87)
Post-mortem interval (h)	39 ± 4.9	43 ± 5.4	38 ± 5.2	49 ± 8.2	39 ± 3.5	46 ± 4.6
(Range)	(25–72)	(18–76)	(10–70)	(22–97)	(10–72)	(18–97)
Brain pH	6.63 ± 0.11	6.42 ± 0.07	6.38 ± 0.12	6.36 ± 0.08	6.52 ± 0.08	6.40 ± 0.05
(Range)	(6.2–7.71)	(6.05–6.72)	(5.9–6.97)	(6.04–6.8)	(5.9–7.71)	(6.04–6.8)

Values are means ± SEM (and ranges). There are no significant demographic differences between diagnostic groups in the Oxford, London or combined series. Tissue was not available from all subjects for every experiment.

### Tissue collection and processing

Blocks were collected from the DPFC (Brodmann area 9/46), cerebellum, hippocampus and (for DAO only) from midbrain containing the substantia nigra. Blocks from the Oxford series were dissected directly at autopsy and stored at −80 °C. Blocks from the London series were dissected from previously snap-frozen coronal brain slices at −20 °C and returned to storage at −80 °C. Sections from both series were cut at 18 µm, collected on Superfrost Plus slides (BDH Merck, Poole, UK) and stored at −80 °C. From some subjects in the Oxford series, formalin-fixed paraffin-wax-embedded blocks were also taken for immunohistochemistry. From these, 10-µm sections were cut and collected on coated slides. All material was coded and experiments carried out blind to diagnosis.

### Generation of antisera to DAO

A rabbit polyclonal antibody against a C-terminal human DAO sequence (peptide H-CGRILEEKKLSRMPPSHL-OH; N-terminal Cys added for coupling) was produced by Cambridge Research Biochemicals (Billingham, UK). Crude antiserum was purified by affinity chromatography on Thiopropyl Sepharose 6B derivatized with the antigen. The concentration of anti-DAO antibody in glycine eluates was 0.77 mg/mL. Specificity to DAO was tested by Western blotting using human tissue lysates (protein medleys, Clontech, VWR International) and recombinant N-terminally flag-tagged human DAO expressed in HEK293 cells. HEK293 cells were cultured in Minimum Essential Medium (MEM, Gibco) supplemented with 10% fetal calf serum and non-essential amino acids (Gibco) at 37 °C and 10% CO_2_. Cells were plated 1 day before transfection at 3.5 × 10^6^ cells per 10-cm plate and transiently transfected with 6 µg plasmid-DNA using Fugene 6 transfection reagent (Roche) according to the manufacturer's instructions. Cells were incubated with transfection mix for 38 h prior to extraction of cell lysates for Western blotting analysis.

### Western blotting

Frozen human and rat tissue sections were homogenized in suspension buffer as described (Eastwood *et al*., 2001b). HEK293 cell lysates were extracted in suspension buffer (50 mm Tris-HCl, pH 7.5, 150 mm NaCl, 10% glycerol, 1 mm EDTA, 0.8% Triton X-100) with added ‘Complete’ protease inhibitors (Roche) and Phosphatase inhibitor cocktail (Sigma). Immediately before electrophoresis samples were mixed 4 : 1 with 5× loading buffer [final concentration 0.1 m Tris-chloride, 2% sodium dodecyl sulphate (SDS), 10% glycerol, 1%β-mercaptoethanol, 0.02% Bromophenol blue] and boiled for 10 min. Clontech protein medleys were diluted in 1× loading buffer (as above, but containing 50 mm DTT instead of β-mercaptoethanol) and boiled for 5 min prior to electrophoresis. Equal protein concentrations and a molecular weight marker rainbow ladder (GE Healthcare, Buckinghamshire, UK or Bio-Rad, Hertfordshire, UK) were fractionated in duplicate by electrophoresis on a 12% SDS/polyacrylamide gel and transferred onto a polyvinyl difluoride membrane. Membranes were immersed in blocking buffer (2% non-fat milk, 0.1% Tween^20^ in phosphate buffered saline (PBS)) for 1 h followed by an overnight incubation at 4 °C with primary antibody [SRR 1 : 500; BD Biosciences; DAO (0.25–0.75 µg/mL); anti-flag M2 1 : 15000; Sigma] in the same buffer. For quantitative measurements, membranes were simultaneously incubated with a commercially available antibody to cyclophilin (1 : 100 000; Abcam, Cambridge, UK), which detects a band of 20 kDa to serve as a loading control. For Western blots with Clontech protein medleys, blots were stripped of the primary anti-DAO antibody (Re-Blot Plus Western blot recycling kit, Chemicon, Hampshire, UK) and re-probed with anti-actin as a loading control (1 : 10000; Chemicon). Membranes were washed three times in blocking buffer, incubated for 1 h with HRP-conjugated secondary antibody in blocking buffer (anti-mouse immunoglobulin 1 : 1000, DAKO, Cambridgeshire, UK, for SRR, flag-M2 and β-actin, and anti-rabbit immunoglobulin 1 : 5000, Chemicon, for DAO and cyclophilin) followed by washing three times in PBS containing 0.1% Tween^20^. Immunoreactive bands were visualized on X-ray film using the ‘ECL Plus’ kit (GE Healthcare) on Kodak BioMax X-Omat AR film (Sigma). Bands were analysed by densitometry and optical density values for the target immunoreactive bands normalized to those of the housekeeping gene signals. Specificity for all antibodies in Western blotting was assessed in control experiments where the primary antibody was omitted. For DAO, a preadsorption control with the antigenic peptide was also carried out.

### Immunohistochemistry

The paraffin-wax-embedded sections were de-waxed in xylene overnight, rehydrated in graded ethanol, and microwaved in antigen unmasking solution (Vector Laboratories, Peterborough, UK) at 100 °C for 10 min. The tissue was pretreated with 3% hydrogen peroxide. Following the blocking of non-specific binding sites in 10% normal horse (SRR) or goat (DAO) serum in PBS-0.3% Triton X-100 (PBS-T), sections were washed in PBS-T and incubated overnight at 4 °C with primary antibody. Primary antibody concentrations were titrated in pilot experiments for optimal detection and used as follows: SRR (BD Biosciences, Oxford, UK) 1 : 35; DAO 1.5 µg/mL, except for DPFC (3.75 µg/mL). After a 30-min treatment with 1 : 100 dilution of biotinylated horse anti-mouse (SRR) or goat anti-rabbit (DAO) immunoglobulin (Sigma), the antibody was localized by the avidin–biotin–peroxidase technique using the Vectastain Elite ABC kit (Vector Laboratories). The peroxidase reaction was demonstrated with 3,3′-diaminobenzidine (DAB). Cresyl violet was used as a counterstain for some sections. Specificity for all antibodies in immunohistochemistry experiments was assessed in control experiments where the primary antibody was omitted and for DAO with a preadsorption control.

### In situ *hybridization histochemistry*

Amplified human DAO cDNA (400-bp fragment, base-pairs 441–840 of Accession No. NM_001917) was subcloned into pGEM-T Easy Vector (Promega, Southampton, UK) following the manufacturer's recommendations. Protocols for riboprobe production and *in situ* hybridization histochemistry have been previously described (Burnet *et al*., 1994). Briefly, riboprobes were produced by linearizing the DAO-pGEM-T construct with appropriate restriction endonucleases and transcribing using either SP6 or T7 promoter sites in the presence of [^35^S]UTP (1000 C_i_/mmol). The probe was diluted with hybridization buffer [75% formamide, 3× standard saline citrate (SSC), 1× Denhardt's solution, 50 mm sodium phosphate buffer, pH 7.4, 10% dextran sulphate, 0.1 mg/mL sheared salmon sperm DNA and 20 mm dithiothreitol] to a final activity of 1.2 × 10^4^ c.p.m./mL. The diluted probe (200 µL) was added to each slide of human tissue sections and covered with a glass coverslip. All slides were incubated overnight at 50 °C in lidded Perspex trays lined with filter paper soaked with 3× SSC/75% formamide.

For post-hybridization washing, slides were first rinsed in 2× SSC, incubated in RNase A (20 µg/mL) for 30 min at room temperature, and immersed in 2× SSC at 55 °C for 10 min, 0.5× SSC at 55 °C for 60 min and finally 0.5× SSC at room temperature for 10 min. Slides were rinsed in ddH_2_O, dried and apposed to X-ray film (Kodak) at room temperature, or dipped in photographic emulsion using standard methods (Eastwood *et al*., 2001b). Emulsion-dipped slides were developed and counterstained with cresyl violet.

### mRNA extraction and real-time PCR

Frozen human tissue sections (DPFC and cerebellum) were homogenized in TRI reagent (Sigma) according to the manufacturer's instructions. Total RNA concentration was determined spectrophotometrically. Total RNA was treated with RNase-free DNase (Promega) according to the manufacturer's instructions. DNase-treated total RNA (1–2 µg) was reverse transcribed using a high-capacity cDNA archive kit (Applied Biosystems, Warrington, UK) and cDNA stored at −70 °C.

Real-time PCR was performed in an ABI Prism 7000 Sequence Detector system (Applied Biosystems) using a real-time PCR kit for SYBR Green I (Eurogentec, Southampton, UK) and 100 nm of forward and reverse primers (Sigma) (SRR (F) AACCTAGTGATGAGTCCAGAGAAA, (R) CTGGTTGGGATGTACCATGA; DAO (F) CGCAGACGTGATTGTCAACT, (R) GGATGATGTACGGGGAATTG; r18s (F) GTAACCCGTTGAACCCCATT, (R) CCATCCAATCGGTAGTAGCG; GAPDH (F) AAGGTGAAGGTCGGAGTC, (R) GAAGATGGTGATGGGATTTC). The primers for DAO, SRR and the ‘housekeeping genes’ ribosomal 18 s (r18s) and glyceraldehyde-3-phosphate dehydrogenase (GAPDH) were designed using Primer 3 software (http://frodo.wi.mit.edu/). Specificity of primers for each transcript was confirmed using a nucleotide–nucleotide alignment search (http://www.ncbi.nlm.nih.gov/BLAST/). Thermal cycling was initiated with incubation at 50 °C for 2 min and 95 °C for 10 min followed by 40 cycles of 95 °C for 15 s, 60 °C for 1 min and a melt curve analysis was performed at the end of each PCR experiment. All reactions were run in triplicate. Pilot studies established reaction efficiency and assay linearity with each primer pair and melt curve analysis confirmed specific product formation with each primer pair. RNA samples which had not been reverse transcribed (negative controls) were subjected to PCR in the same assay as cDNA samples.

Fluorescence emission intensity from SYBR Green I was plotted against Ct (cycle of threshold) values corresponding to the number of PCR cycles. For each sample the Ct value was measured at a given threshold of fluorescence emission intensity corresponding to the exponential phase of the PCR reaction. The abundance of target cDNA in each sample was calculated from a standard curve and normalized to the levels of r18s and GAPDH in the same sample.

### Antipsychotic treatment in rats

Adult male Sprague–Dawley rats (Harlan-Olac, Bicester, UK) weighing 225–250 g were treated with the conventional antipsychotic haloperidol (1 mg/kg/day; *n* = 8) or the same volume of saline (*n* = 8) by intraperitoneal injection once daily for 14 days, killed and brain tissue processed as described (Law *et al*., 2004). All procedures were carried out in accordance with UK Home Office Guidelines. Protein was extracted from frozen brain sections and used for Western blotting as described.

### Statistical analysis

Previous studies showed that the detected abundance of some mRNAs and proteins differed between the two brain series (Eastwood *et al*., 2000, 2001a, b). In case a similar effect was seen here, *Z*-scores were computed (Eastwood *et al*., 2001b) to allow data from the series to be combined for analysis. We also inspected data from each series separately (see Results). The effects of potential confounding variables (brain pH, post-mortem interval and age) were explored using correlations partialled for diagnosis. Effects of diagnosis were investigated with one-way analysis of variance (anova) with brain pH, post-mortem interval and age as covariates where the correlation was significant (*P <* 0.05). For data not normally distributed, the Mann–Whitney *U*-test was used. To test for an effect of antipsychotic drug treatment in rats, an independent samples *t*-test was used.

## Results

### Validation of DAO and SRR antibodies

**Fig. 1:**
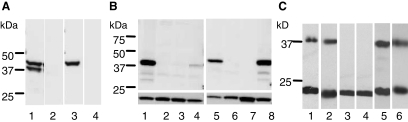
Detection of DAO and SRR. (A) Validation of DAO antibody using recombinant flag-DAO expressed in HEK293 cells. Detection with anti-DAO (lanes 1 and 2) and anti-flag (lanes 3 and 4). Flag-DAO is detected as a ∼42-kDa band with anti-DAO (lane 1) and anti-flag (lane 3) in flag-DAO transfected HEK293 cells but not mock transfected cells (lanes 2 and 4). Expressed untagged DAO is detected as a ∼39-kDa band, of predicted size for DAO, with anti-DAO antibody (lane 1 lower band) but not anti-flag (lane 3). (B) Validation of DAO antibody using human tissue samples (Clontech protein medleys); lane 1, cerebellum; lane 2, amygdala; lane 3, hippocampus; lane 4, thalamus; lane 5, spinal cord; lane 6, lung; lane 7, heart; lane 8, kidney. A specific signal for DAO was detected at ∼39 kDa in the cerebellum, spinal cord and kidney. Blots were re-probed with anti-β-actin as a loading control (lower panel). (C) Representative Western blots of DAO (lanes 1 and 2), DAO pre-adsorption control (lanes 3 and 4), and SRR (lanes 5–6) in protein extracts of post-mortem cerebellar tissue from human (lanes 1, 3 and 5) and rat (lanes 2, 4 and 6). DAO was detected at ∼39 kDa and SRR at ∼38 kDa. Blots were simultaneously probed for cyclophilin as a loading control (lower band), which was detected at ∼20 kDa.

A novel anti-DAO antibody, directed against a C-terminal human DAO peptide, was validated by Western blotting using lysates from HEK-293 cells over-expressing an N-terminally flag-tagged human DAO construct, containing a start codon in the flag tag as well as the DAO start codon. The antibody recognized recombinant DAO as a double band at ∼39 and ∼42 kDa (Fig. 1A, lane 1). The lower band, of predicted molecular weight for DAO (Gavazzi *et al*., 1987; Katagiri *et al*., 1991; Moreno *et al*., 1999), probably represents transfected untagged DAO translated from the DAO start codon, as only the higher band, corresponding to flag-tagged DAO (translated from the flag start codon), was detected with an anti-flag antibody (Fig. 1A, lane 3) and HEK-293 cells do not express endogenous DAO (mock-transfected cells; Fig. 1A, lanes 2 and 4).

In order to confirm that the anti-DAO antibody could detect endogenous DAO in human tissue samples, human protein medleys from different brain regions and peripheral tissues were analysed by Western blotting. A strong single band at approximately ∼39 kDa was detected in tissues reportedly expressing high levels of DAO (cerebellum, spinal cord and kidney; Katagiri *et al*., 1991; Horiike *et al*., 1994) (Fig. 1B). Anti-DAO detected a similar band in our extracts of frozen human and rat cerebellar tissue (Fig. 1C, lanes 1–2), and preadsorption of the DAO antibody with antigenic peptide abolished this band (Fig. 1C, lanes 3–4). In human DPFC extracts, detection of DAO by Western blotting was extremely variable and sometimes absent, despite trying a range of antibody and protein concentrations (data not shown). Similarly, in rat frontal cortex, DAO could not be detected by Western blotting (data not shown).

SRR was detected as a single band of predicted molecular weight, ∼38 kDa (Wolosker *et al*., 1999b; De Miranda *et al*., 2000), in human and rat cerebellum (Fig. 1C, lanes 5–6) and in human DPFC and rat prefrontal cortex (data not shown). Omission of primary antibody abolished SRR immunoreactivity (data not shown).

The housekeeping genes cyclophilin and β-actin were detected as single bands of predicted molecular weights of ∼20 and ∼43 kDa, respectively (Fig. 1B and C).

**Fig. 2:**
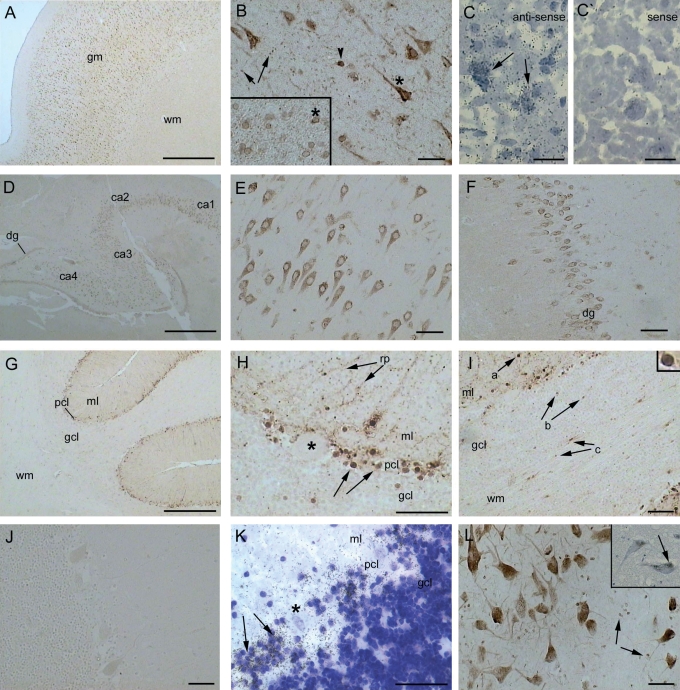
Regional and cellular distribution of DAO in the human brain. (A) DAO immunoreactivity in the DPFC demonstrating an enhancement of labelling in the grey matter (gm) compared with the white matter (wm). (B) DAO immunoreactivity in DPFC grey matter demonstrating immunolabelling of pyramidal neurons (*), throughout their cell bodies, and sometimes granular. Punctate spots of immunolabelling are seen throughout the neuropil (arrows), in addition to the labelling of occasional small round cells (arrowhead). Inset: DAO immunoreactivity in the white matter as punctate spots of fibre labelling and moderate staining of numerous small round cells, often with peri-cellular enhancement (*). (C) Emulsion dipped antisense *in situ* hybridized DPFC sections showing localization of DAO mRNA to pyramidal cells (arrows). (C′) Corresponding image after sense probe hybridization. (D) DAO immunoreactivity in the hippocampus, demonstrating labelling predominantly in neuronal subfields ca1–4 and (in some cases) in the granule cells of the dentate gyrus (dg). (E) DAO immunoreactivity in ca1 pyramidal neurons. (F) Dentate gyrus, showing moderate staining of granule cells (dg). (G) Cerebellar DAO immunoreactivity showing localization predominantly to the Purkinje cell layer (pcl) with moderate immunolabelling in the molecular layer (ml), granule cell layer (gcl) and white matter (wm). (H) DAO immunoreactivity in the cerebellum demonstrating strongly immunopositive putative Bergmann glia (arrows) in the Purkinje cell layer (pcl) with unstained Purkinje cells (*). In the molecular layer (ml) moderate immunolabelling of putative Bergmann glia radial processes (rp) can be seen. (I) Cerebellar DAO immunoreactivity localized to small round cells of similar size in the molecular layer (arrow a), granule cell layer (arrow b) and white matter (arrow c). Often these cells showed peri-cellular enhancement in staining (inset). (J) Pre-adsorption control for DAO immunohistochemistry in the cerebellum. (K) Emulsion dipped antisense *in situ* hybridized sections demonstrating silver grains clustered over putative Bergmann glia (arrows) but not over Purkinje cells (*) or the granule cell layer (gcl). (L) DAO immunoreactivity in the substantia nigra pars compacta localized to large neurons and to some small round cells, putatively glia (arrows). Cresyl violet-stained sections (inset) demonstrate lipofuscin in neurons (arrow). Scale bars = 1 mm (A), 0.5 mm (D, G), 50 µm (B, C, C′, E, F, H–L).

**Fig. 3:**
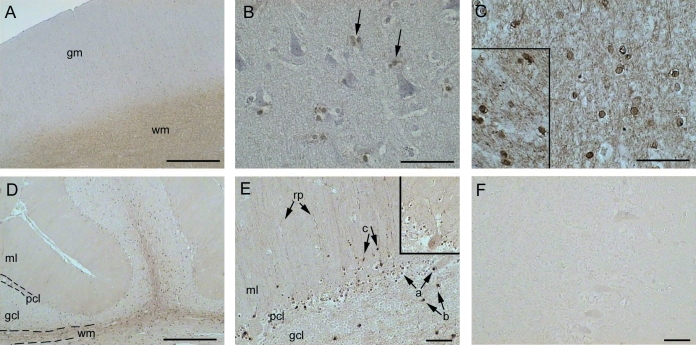
Regional and cellular distribution of SRR immunoreactivity in the human brain. (A) DPFC, demonstrating an enhancement of staining in white matter (wm) over grey matter (gm). (B) SRR immunoreactivity with cresyl violet counterstaining in DPFC grey matter, demonstrating immunolabelling of putative glia (arrows) amongst immunonegative pyramidal neurons (purple). (C) SRR immunoreactivity in the DPFC white matter localized to putative glia and as punctate white matter labelling. Inset: high-power image of SRR immunoreactivity in the hippocampal white matter. (D) SRR immunoreactivity in the cerebellum in the white matter (wm), granule cell layer (gcl), Purkinje cell layer (pcl) and molecular layer (ml). (E) SRR immunoreactivity in the cerebellum localized to Bergmann glia of the Purkinje cell layer (arrows a) and their radial processes (rp) in the molecular layer, putative glia of the granule cell layer (arrows b) and putative glia of the molecular layer (arrows c). Purkinje cells were occasionally moderately immunolabelled (inset). (F) Control section after omission of primary antibody. Scale bars = 1 mm (A), 0.5 mm (B), 50 µm (C–F).

In immunohistochemistry experiments, omission of the SRR primary antibody, and preadsorption of the DAO antibody with the antigenic peptide, abolished immunolabelling (see below; Figs 2J and 3F).

### Regional and cellular expression of DAO in the human brain

DAO was detected by immunohistochemistry in all regions examined, but the cell types and staining intensity differed between regions.

#### DPFC

In the DPFC grey matter, DAO immunoreactivity was predominantly neuronal (Fig. 2A), and visible throughout the cytoplasm of pyramidal neuron cell bodies, sometimes granular, and surrounding unstained nuclei (Fig. 2B). Granular immunostaining may reflect peroxisomal DAO (Arnold *et al*., 1979; Moreno *et al*., 1999). Punctate spots of immunoreactivity were also visible throughout the neuropil (Fig. 2B). Immunolabelling of small round cells was visible in some cases (Fig. 2B); given their size they may be glia, or, alternatively, interneurons. In DPFC white matter, immunostaining was weak, but moderate punctate DAO immunoreactivity was visible in addition to the labelling of small round cells, sometimes demonstrating a peri-cellular enhancement in their staining (Fig. 2B, inset). Again the exact identity of these cells is unknown, but based on their size, morphology and location, they are putatively identified as glia.

*In situ* hybridization and emulsion dipping of DPFC sections revealed DAO mRNA signals clustered over pyramidal neurons with minimal labelling of sense control sections (Fig. 2C and 2C′). In the white matter DAO mRNA signals were marginal (data not shown).

#### Hippocampus

DAO immunoreactivity in the hippocampus was principally localized to the stratum pyramidale of Ammon's horn and in some cases to the dentate gyrus granule cell layer (Fig. 2D). Pyramidal neurons in CA1 displayed moderate to intense staining (Fig. 2E). CA2/CA3 and CA4 regions displayed moderate neuronal labelling but staining appeared less intense than in CA1 (data not shown). DAO immunoreactivity was visible throughout pyramidal cell bodies, sometimes granular or peri-cellular, while the nucleus was unlabelled (Fig. 2E). In the dentate gyrus, immunoreactivity was variable; half the cases studied demonstrated moderate to strong immunoreactivity of granule cells (Fig. 2F), while the remainder demonstrated marginal or no immunostaining (data not shown).

*In situ* hybridization and emulsion dipping of hippocampus sections revealed DAO mRNA signals clustered over pyramidal neurons in the CA subfields, while signals in other regions were low (data not shown).

#### Cerebellum

DAO immunoreactivity in the cerebellum was localized predominantly to the Purkinje cell layer (Fig. 2G), and therein to small round cells, which, given their size and location, are putative Bergmann glia (Fig. 2H). The Purkinje cells themselves were unlabelled (Fig. 2H). Processes traversing the molecular layer were also immunoreactive, some of which appeared to arise from Bergmann glia, putatively their radial processes (Fig. 2H). The molecular layer also demonstrated occasional immunolabelling of small round cells (Fig. 2I). In the granule cell layer, Golgi cells and granule cells were unlabelled, but scattered small round cells, somewhat larger than granule cells, were immunoreactive (Fig. 2I). Cells of the white matter, with a similar shape and size to those labelled in the molecular and granule cell layers, were also immunoreactive (Fig. 2I). While the specific identity of these molecular, granule cell and white matter layer cells is unknown, their size, morphology and location suggest they are glia, although the molecular layer cells could also be basket/stellate cells. At the subcellular level, some moderately stained cells, particularly those in the granule cell layer, demonstrated a peri-cellular enhancement of immunolabelling (Fig. 2I, inset).

*In situ* hybridization and emulsion dipping of cerebellar sections revealed DAO mRNA signals primarily clustered over small round cells in the Purkinje cell layer, putatively Bergmann glia (Fig. 2K). There were few silver grains over the Purkinje cells themselves (Fig. 2K).

#### Substantia nigra pars compacta

In midbrain sections, cresyl violet staining revealed large, putatively dopaminergic, neurons containing melanin pigment within the substantia nigra pars compacta (Fig. 2L, inset). DAO immunoreactivity was strong in these neurons, and, in cases with stronger immunostaining, immunolabelling of putative glia was also observed (Fig. 2L).

### Regional and cellular expression of SRR in the human brain

#### DPFC

SRR immunolabelling in the DPFC was more abundant in the white matter compared with the grey matter (Fig. 3A). In the grey matter, immunolabelled small round cells were observed amongst unlabelled cortical pyramidal neurons (Fig. 3B). In the white matter, immunolabelling of similar small round cells, more prevalent than those in the grey matter, was observed in addition to punctate white matter labelling (Fig. 3C). These cells frequently demonstrated a punctate or peri-cellular enhancement in immunostaining (Fig. 3C). Based on their size, location and morphology they are putatively identified as glia.

#### Hippocampus

Similar to the DPFC, SRR immunoreactivity in the hippocampus was predominantly localized to the white matter, including the alveus, and concentrated over small round cells in addition to punctate immunostaining (Fig. 3C, inset).

#### Cerebellum

SRR immunoreactivity in the cerebellum was seen in all layers (Fig. 3D). In the Purkinje cell layer, small round cells situated between Purkinje cells were strongly immunolabelled (Fig. 3E). Their size and location suggest they are putative Bergmann glia. The Purkinje cells were occasionally moderately immunolabelled (Fig. 3E, inset). In the molecular layer, there was moderate immunolabelling of putative Bergmann glia radial processes; additionally, small round cells, potentially basket/stellate or glial cells, were immunolabelled (Fig. 3E). Throughout the granule cell layer, scattered strongly immunopositive cells, slightly larger than granule cells, were observed (Fig. 3E). No cells with an appearance suggestive of Golgi cells were labelled. In the white matter, there was abundant and strong immunostaining of small round cells (data not shown). The immunolabelled cell types in these layers were observed to have a peri-cellular enhancement in immunolabelling (data not shown) and are presumed to be glia. The strong immunoreactivity of the cerebellar white matter also arose from punctate immunostaining (data not shown).

### DAO expression in schizophrenia

**Fig. 4:**
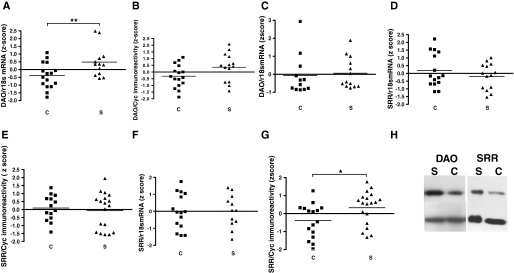
DAO and SRR expression schizophrenia. (A) Real-time PCR analysis of DAO mRNA normalized to r18s in the cerebellum. **P < 0.005. (B) Western blot analysis of DAO immunoreactivity normalized to cyclophilin (Cyc) in the cerebellum. (C) Real-time PCR analysis of DAO mRNA normalized to r18s in the DPFC. (D) Real-time PCR analysis of SRR mRNA normalized to r18s in the cerebellum. (E) Western blot analysis of SRR immunoreactivity normalized to cyclophilin (Cyc) in the cerebellum. (F) Real-time PCR analysis of SRR mRNA normalized to r18s in the DPFC. (G) Western blot analysis of SRR immunoreactivity normalized to cyclophilin (Cyc) in the DPFC. *P < 0.05. (H) Representative Western blots for DAO in the cerebellum and SRR in the DPFC (upper bands) of patients with schizophrenia (S) and control subjects (C). The lower band shows the loading control cyclophilin.

Cerebellar DAO mRNA levels normalized to r18s correlated with brain pH (*P =* 0.048). No other effects of brain pH, age or post-mortem interval were seen. DAO mRNA was increased in patients with schizophrenia compared with control individuals whether normalized to r18s (*F*_1,26_ = 10.01, *P* = 0.004) (Fig. 4A) or GAPDH mRNA (data not shown). DAO immunoreactivity in the cerebellum showed a trend to being increased in schizophrenia (*F*_1,28_ = 3.79, *P* = 0.062) (Fig. 4B and H).

In the DPFC, DAO mRNA normalized to r18s was unchanged in schizophrenia (Mann–Whitney *U*-test, *P* = 0.33) (Fig. 4C) as was DAO mRNA normalized to GAPDH (data not shown). In the DPFC, because DAO immunoreactivity was extremely variable and most subjects expressed levels of DAO protein undetectable by Western blotting, no quantification was attempted.

The direction of change in DAO mRNA and protein in the cerebellum in schizophrenia was consistent between the two brain series, but the increase in schizophrenia appeared more marked in the London series (Table 2).

**Table 2:** DAO and SRR expression in the DPFC and CB in schizophrenia divided by origin of tissue

	Oxford series		London series	
		
	Controls	Patients	Controls	Patients
DAO	(*n* = 6–9)	(*n* = 7–8)	(*n* = 6–7)	(*n* = 6)
mRNA				
CB	7.65 ± 1.11	9.69 ± 1.43	11.74 ± 0.86	15.97 ± 1.29
DPFC	8.70 ± 2.77	6.99 ± 0.90	4.335 ± 2.52	9.38 ± 3.69
Protein				
CB	0.53 ± 0.06	0.60 ± 0.08	1.96 ± 0.44	3.40 ± 0.47
DPFC	n.d.	n.d.	n.d.	n.d.
SRR	(*n* = 6–8)	(*n* = 6–10)	(*n* = 7–9)	(*n* = 6–9)
mRNA				
CB	1.18 ± 0.33	1.02 ± 0.21	1.10 ± 0.16	0.89 ± 0.15
DPFC	1.02 ± 0.13	0.71 ± 0.17	0.95 ± 0.16	1.27 ± 0.10
Protein				
CB	0.63 ± 0.08	0.70 ± 0.09	0.79 ± 0.05	0.66 ± 0.07
DPFC	0.68 ± 0.08	0.88 ± 0.06	0.48 ± 0.06	0.59 ± 0.07

Values are means ± SEM. mRNA normalized to r18s; protein normalized to cyclophilin. n.d., not determined.

### SRR expression in schizophrenia

No effects of age, brain pH or post-mortem interval were seen on SRR mRNA and protein measurements. In the cerebellum, SRR mRNA was unchanged in schizophrenia, whether normalized to r18s (*F*_1,27_ = 1.05, *P* = 0.32) (Fig. 4D) or GAPDH mRNA (data not shown). SRR immunoreactivity in the cerebellum was also unchanged in schizophrenia (*F*_1,30_ = 0.22, *P* = 0.64) (Fig. 4E). In the DPFC, SRR mRNA was unchanged in schizophrenia whether normalized to r18s (*F*_1,25_ = 0.002, *P* = 0.96) (Fig. 4F) or GAPDH mRNA (data not shown). SRR immunoreactivity in the DPFC was increased in schizophrenic patients compared with control individuals (Mann–Whitney *U*-test, *P* = 0.027) (Fig. 4G and H). This effect was consistent between the Oxford and London series (Table 2).

### DAO and SRR protein in the rat brain following haloperidol administration

DAO immunoreactivity, in the rat cerebellum was unaffected by haloperidol (saline = 1.27 ± 0.14 (mean ± SEM), haloperidol = 1.58 ± 0.35, *t*_(14)_ = −0.817, *P* = 0.428). Values were normalized to cyclophilin immunoreactivity. We could not detect DAO immunoreactivity in the frontal cortex. Haloperidol had no effect on SRR immunoreactivity, normalized to cyclophilin, in the cerebellum (saline = 1.56 ± 0.15, haloperidol = 1.60 ± 0.16, *t*_(13)_ = −0.169, *P* = 0.869) or frontal cortex (saline = 0.83 ± 0.04, haloperidol = 0.84 ± 0.06, *t*_(14)_ = −0.147, *P* = 0.885).

## Discussion

This study investigated the regional and cellular expression in human brain of the enzymes responsible for the degradation (DAO) and synthesis (SRR) of d-serine. It also examined whether the expression of these enzymes was altered in the DPFC or cerebellum in schizophrenia, a disorder in which both enzymes have been implicated as part of a broader abnormality in NMDA receptor-mediated signalling. Our main findings are that: (1) DAO and SRR are expressed widely in adult human brain, in discrete but overlapping cell populations (Table 3). DAO localized to glia in the cerebellum, but in the cerebral cortex was predominantly neuronal. In all regions examined, SRR immnuoreactivity was principally glial, and was more abundant in white matter. (2) In schizophrenia, DAO mRNA was increased in the cerebellum, and SRR increased in the DPFC. These findings have implications for our understanding of the cellular basis of d-serine metabolism and for the roles of DAO and SRR in the normal brain, as well as for their involvement in schizophrenia.

**Table 3:** Cellular profile of DAO and SRR immunoreactivity in the human brain

	DAO	SRR
Cerebellum		
Purkinje cells	0	+/–
Molecular layer cells*	+	+
Granule cells	0	0
Golgi cells	0	0
Bergmann glia	+++	+++
Other glia	++	+++
Molecular layer neuropil	+	+
Granule cell layer neuropil	+/–	+/–
White matter neuropil	+/–	++
DPFC		
Pyramidal neurons	+++	0
Grey matter glia	+	++
White matter glia	+	+++
Grey matter neuropil	+	+/–
White matter neuropil	+	+++
Hippocampus		
Dentate gyrus granule cells	+/–	0
Pyramidal neurons	+++	0
Glia	+/–	++
Grey matter neuropil	0	+/–
White matter neuropil	0	+++
Substantia nigra pars compacta		
Dopamine neurons	+++	n.d.
Glia	+/–	n.d.
Neuropil	0	n.d.

Immunoreactivity: 0, absent; +/–, weak and variable; +, weak; ++, moderate; +++, strong; n.d., not determined.

* Stellate/basket or glia.

### DAO expression in human brain

DAO immunoreactivity and mRNA were robustly detected in the cerebellum, in keeping with previous studies of its expression and activity in human (Kapoor *et al*., 2006) and rodent cerebellum (Katagiri *et al*., 1991; Horiike *et al*., 1994; Schell *et al*., 1995; Moreno *et al*., 1999; Wang & Zhu, 2003; Yoshikawa *et al*., 2004b). Cerebellar DAO expression, both mRNA and immunoreactivity, was primarily localized to Bergmann glia (Fig. 2H). Immunoreactivity also localized to other small round cells scattered in the molecular, granule cell and white matter layers that we assume are glia (Fig. 2I). These observations are consistent with previous immunocytochemical (Moreno *et al*., 1999) and histochemical (Horiike *et al*., 1987) findings in the rat brain. Moreno *et al*. (1999) also reported DAO immunoreactivity in Golgi and Purkinje cells, which we did not observe in the human cerebellum. This may be due to a species difference in DAO expression, as is seen for other aspects of DAO biology (Pollegioni *et al*., 2007). Alternatively, the discrepancy may be attributable to the antibody used by Moreno *et al*. (1999), which subsequent data suggest may cross-react with d-aspartate oxidase (Shleper *et al*., 2005), itself abundantly expressed in Golgi and Purkinje cells of the rat (Zaar *et al*., 2002).

The situation regarding DAO in the forebrain has been less clear. Our data confirm that DAO is unequivocally expressed, both as mRNA and as protein, in the human cerebral cortex (DPFC and hippocampus), and also show that this is mainly neuronal with a small glial contribution, consistent with the immunocytochemical findings of the rat study by Moreno *et al*. (1999), but unlike the situation in cerebellum. These findings raise three issues worthy of mention. First, our antibody did not reliably detect DAO protein by Western blotting in DPFC (or in hippocampus), even though it did in cerebellum, but worked reliably for immunohistochemistry in all areas. Its specificity for DAO is not questioned by this failure, given the experimental controls and corroboration of the immunohistochemical data in DPFC by our *in situ* hybridization findings, but it does require explanation. Most likely, there is a regional difference in the antigenicity of DAO, possibly consequent to differences in post-translational modification or conformation, which is differentially affected by Western blotting and immunohistochemical protocols. Whether this is related to the fact that cerebellum and cerebral cortex predominantly express DAO in different cell types (glia vs. neurons), or the fact that cerebellum contains a DAO transcript variant not detectable in cortex (Kapoor *et al*., 2006), is unknown. Of note, Bendikov *et al*. (2007) did robustly detect DAO by Western blotting in the human parietal cortex, using an antibody raised against purified hog DAO, whereas ours was generated against a specific peptide sequence. Potentially, their antibody may have been less susceptible to any inter-regional differences in DAO sequence, conformation or structure.

The second issue relates to the fact that cortical DAO immunoreactivity was mainly neuronal, whereas it was largely, if not exclusively, glial in the cerebellum. The basis for, and significance of this is unclear. In the DPFC, it implies that much d-serine catabolism is neuronal rather than glial, consistent with the recent observations that the major d-serine transporter, Asc-1, is also predominantly neuronal (Helboe *et al*., 2003; Matsuo *et al*., 2004; Rutter *et al*., 2007). Together with the localization of SRR (Table 3), the findings suggest that, in the cortex, d-serine is synthesized mainly in glia, but degraded in neurons.

The third question concerns why cortical DAO expression is robust, but cortical DAO activity is virtually undetectable (Katagiri *et al*., 1991; Horiike *et al*., 1994; Schell *et al*., 1995; Wang & Zhu, 2003; Kapoor *et al*., 2006). One possibility is that cortical DAO is inactive, perhaps a remnant from an earlier, developmental function, although its robust prolonged expression argues against this. Or, perhaps cortical DAO has a substrate other than d-serine, and catalyses a reaction not detected by the commonly used functional assay. However, if this were the case, it would raise the alternative question of how d-serine is degraded in the cortex.

DAO immunoreactivity was strong in putative dopaminergic neurons of the substantia nigra pars compacta, consistent with an observation in the rat (Moreno *et al*., 1999). This finding is of clinical significance given the demonstration that DAO can oxidize d-DOPA in an alternative pathway for dopamine synthesis, and the suggestion that d- and l-DOPA may be combined therapeutically for the treatment of Parkinson's disease (Brannan *et al*., 1996; Moses *et al*., 1996; Wu *et al*., 2006; Kawazoe *et al*., 2007). However, the presence of endogenous d-DOPA or other d-isomers of catecholamine precursors in mammals is, to our knowledge, unknown. Nevertheless, the presence of DAO in these neurons suggests that it may play a role in dopaminergic function and, speculatively, the involvement of dopamine or dopamine–glutamate interactions in schizophrenia (Laruelle *et al*., 2003).

### SRR expression in human brain

SRR expression was detected in the DPFC and hippocampus, consistent with previous studies in rodent (Wolosker *et al*., 1999a; Wang & Zhu, 2003; Yoshikawa *et al*., 2004a; Kartvelishvily *et al*., 2006), primate (Xia *et al*., 2004) and human (Steffek *et al*., 2006; Bendikov *et al*., 2007). Within these regions, SRR immunoreactivity was localized to glia of both the grey and the white matter (Fig. 3B and C). Previous reports show that SRR immunolabelled glia are astroctyes, based on colocalization with glial fibrillary acidic protein (Wolosker *et al*., 1999a; Xia *et al*., 2004; Kartvelishvily *et al*., 2006). However, we cannot rule out a microglial (Wu & Barger, 2004) or oligodendrocytic contribution to SRR expression, as a detailed analysis of glial subtype was beyond the scope of this study. Whilst our findings are consistent with a glial localization of SRR, the greater white than grey matter staining in DPFC (and hippocampus) is at odds with Wolosker *et al*. (1999a), who suggest that SRR is enriched in grey matter astrocytes, as is d-serine (Nagata *et al*., 1994; Schell *et al*., 1995, 1997). However, others report abundant d-serine staining in the white matter (Kartvelishvily *et al*., 2006; Williams *et al*., 2006) and d-serine concentrations in the white matter of human brain are equal to or greater than those in grey matter (Kumashiro *et al*., 1995).

We identified SRR mRNA and immunoreactivity in the human and rat cerebellum. Previous studies of cerebellar SRR have been conflicting. Wang & Zhu (2003) could not detect SRR by immunoblotting in adult mouse cerebellum, in line with reported low or negligible levels of rodent cerebellar d-serine (Nagata *et al*., 1994; Hashimoto *et al*., 1995a, b; Schell *et al*., 1995, 1997; Hamase *et al*., 1997; Morikawa *et al*., 2001; Yasuda *et al*., 2001; Wang & Zhu, 2003). However, other studies have robustly detected SRR mRNA (Yoshikawa *et al*., 2004a) and d-serine immunoreactivity (Williams *et al*., 2006) in adult rodent cerebellum. Furthermore, intracerebroventricular administration of l-serine increases cerebellar d-serine levels (Hashimoto, 2002), implying that SRR is present (and active) in the adult cerebellum. Our data indicate that there is a significant and sustained expression of SRR in adult human cerebellum. It remains to be explained therefore why the majority of studies cited above suggest cerebellar d-serine levels are negligible. Possibilities include the SRR being inactive, or that it is catalysing conversion of a substrate other than l-serine, although the implications of the Hashimoto (2002) study would not support this. Another possibility is that SRR is acting as an eliminase enzyme, breaking down d-serine (Foltyn *et al*., 2005). Alternatively, the low d-serine levels seen in cerebellar homogenates may be misleading. The rapid degradation of d-serine by DAO in the cerebellum (Morikawa *et al*., 2001) may in fact show the importance of tight regulation of cerebellar d-serine levels. Moreover, significant d-serine concentrations may occur at localized sites, such as the Bergmann glia (Williams *et al*., 2006), where there is robust SRR expression, and thereby impact on NMDAR-mediated transmission in the vicinity.

A final aspect of SRR distribution that has remained unclear is whether SRR is expressed by neurons. It has been suggested to be exclusively or principally glial, based on studies in the adult primate (Xia *et al*., 2004) and developing rat brain and rat neuronal and glial primary cultures (Wolosker *et al*., 1999a). However, others demonstrate SRR expression in mouse neuronal cultures (Yoshikawa *et al*., 2006) as well as in cortical neurons of both the developing and the adult rat brain (Kartvelishvily *et al*., 2006). We did not identify any unequivocally SRR-immunoreactive neurons in the DPFC or hippocampus. This negative result may reflect a species difference, or could reflect a lack of sensitivity of our SRR antibody under the fixation and processing conditions pertaining in the post-mortem tissue. On the other hand, we did find moderate SRR immunostaining of some cerebellar Purkinje neurons, consistent with a minor neuronal contribution to SRR expression in human brain.

### DAO and SRR expression in schizophrenia

If DAO contributes to NMDAR hypofunction in schizophrenia, then presumably it does so via enhancement (rather than impairment) of DAO function, as this will promote d-serine degradation and thence decreased d-serine occupancy of NMDARs. Our finding of increased DAO mRNA in the cerebellum, without any change in SRR expression, may offer support to this hypothesis, and extends a preliminary study that also found increased cerebellar DAO mRNA in schizophrenia (Kapoor *et al*., 2006). The findings also support the emerging view that the cerebellum is an integral part of the circuitry affected in schizophrenia (e.g. Eastwood *et al*., 2003– for reviews see Katsetos *et al*., 1997; Andreasen *et al*., 1998; Konarski *et al*., 2005). However, our data are subject to the caveat that although DAO mRNA was significantly increased, DAO immunoreactivity showed only a trend increase (*P =* 0.062). Moreover, we did not determine whether DAO activity is elevated. However, it is noteworthy that Kapoor *et al*. (2006) did find enhanced cerebellar DAO activity in schizophrenia to accompany the mRNA elevation. In this regard, were DAO function increased in schizophrenia the consequences would be difficult to predict, given the uncertain status and function of d-serine in the cerebellum outlined above. One possibility, given our demonstration of DAO and SRR in Bergmann glia and unchanged cerebellar SRR in schizophrenia, is that a putative increased DAO could impact Bergmann glia d-serine levels and thence, speculatively, their regulation of synaptic input to Purkinje neurons (Cid & Ortega, 1993; Brockhaus & Deitmer, 2002; Huang & Bordey, 2004).

Increased cerebellar DAO in schizophrenia may arise for one of several reasons. The fact that DAO mRNA is increased indicates that the mechanism is likely to involve transcriptional regulation of DAO expression. This perhaps relates to DAO's candidacy as a putative schizophrenia susceptibility gene, as the associated polymorphisms are all non-coding and so the mechanism of association is probably through an effect on gene expression, as for other risk genes (Harrison & Weinberger, 2005; Law *et al*., 2006). Our sample size was too small to investigate whether any of the schizophrenia-associated DAO polymorphisms contributed to the elevated DAO expression seen here in the disorder. Regardless, as DAO risk alleles are carried by only a minority of cases and by some control subjects as well, this seems unlikely to explain a diagnostic difference in DAO expression. Moreover, as DAO was not increased in the DPFC (present data and Bendikov *et al*., 2007) in schizophrenia, one would have to postulate a region-specific effect of genotype which, although not unprecedented (Law *et al*., 2007), is not parsimonious. Also, DAO genotype does not predict d-serine levels (Yamada *et al*., 2005). Instead, increased cerebellar DAO in schizophrenia may arise as part of the broader pathophysiological processes, e.g. downstream of other genetic, developmental or environmental influences. One example in the last category is anti-psychotic medication. Although there was no significant effect of haloperidol administration in rats on cerebellar DAO expression (*P =* 0.428), the means were ∼24% higher in the treated animals, and so it is possible that medication could have contributed to the elevation seen in patients. An early study reported that the antipsychotic drug chlorpromazine inhibits DAO activity (Yagi *et al*., 1956), but to our knowledge there have been no subsequent investigations of the effects of antipsychotics on DAO expression or activity. Future human post-mortem studies of DAO expression in larger samples with more accurate and extensive medication histories will help to clarify this issue.

SRR immunoreactivity was increased in schizophrenia, but unlike DAO, this occurred in the DPFC not the cerebellum, and was not accompanied by elevated SRR mRNA. The increase was not hypothesized – in that it would suggest increased rather than decreased d-serine levels – and its explanation remains unclear. It may be consequent to other pathophysiological changes; for example, it may reflect an attempted compensatory response to possible disease-associated reductions in cortical d-serine, or that cortical SRR is acting as an eliminase (Foltyn *et al*., 2005), as noted above. This latter explanation has the attraction of thereby explaining how d-serine is metabolized in the cortex despite absent DAO activity; however, it remains controversial as to whether the eliminase reaction occurs under physiological conditions (Strisovsky *et al*., 2005). Mechanistically, the fact that SRR was elevated but not SRR mRNA suggests regulation at the translational or post-translational level. One possibility is decreased protein degradation, as SRR protein levels are regulated by ubiquitin-dependent proteasomal degradation, a mechanism modulated by the SRR binding partner Golga3 (Dumin *et al*., 2006). Whether Golga3 is altered in schizophrenia is unknown.

Speculation as to the significance and the cause of increased DPFC SRR in schizophrenia should in any event be limited, as variable findings have emerged from two other recent studies. First, Steffek *et al*. (2006) reported no change in SRR in prefrontal cortex in schizophrenia, although they did find increased SRR in the hippocampus; the different demographics of the subjects studied (theirs were considerably older) may account for the anatomical discrepancy. In contrast, in a third brain series, Bendikov *et al*. (2007) found reduced SRR in frontal cortex (area 10) and hippocampus. Unlike the other studies, these authors also directly measured d-serine concentrations, overcoming some of the interpretational uncertainty. They replicated the prior reports of reduced cerebrospinal fluid and blood d-serine in schizophrenia, but did not find a reduction in parietal cortex. Overall, therefore, no consensus has yet emerged from the data regarding SRR expression, nor d-serine itself, in the cerebral cortex in schizophrenia.

### Limitations of the study

Some measures of DAO expression differed between the two cohorts of brains studied here (Table 2), even though the basic demographics are similar (Table 1). The reasons are unknown. However, because the effect of origin of brains had been seen previously we conducted our primary data analyses on *Z*-scored data, allowing the two series to be combined, generating a larger data set and thence more robust results. Nevertheless, this variability does raise the issue of the general applicability of the findings; inspection of the raw data shows that the increased cerebellar DAO immunoreactivity and mRNA in schizophrenia, whilst seen in both the London and Oxford series, was more apparent in the former (Table 2). Interestingly, expression of G72 (DAOA) in schizophrenia has also shown a cohort effect with a larger change in schizophrenia in one series than the other (Korostishevsky *et al*., 2004). It is plausible that expression of DAO, and other genes in its regulatory pathway, are particularly susceptible to unknown factors (ranging from genetic variation to peri-mortem confounders) that differ between brain series. The other main limitation of the present study is that d-serine itself was not measured, even though most of the functional implications of altering DAO and SRR expression are implicitly or explicitly the result of their effect upon d-serine levels. In our case, tissue availability precluded measurement of d-serine, and given some reported confounding factors of d-serine measurement in post-mortem tissue (Kumashiro *et al*., 1995), other studies may also have to use the activity and/or expression of genes involved in d-serine metabolism, or its concentration in blood or cerebrospinal fluid (Hashimoto *et al*., 2003, 2005) as proxy measures.

## Conclusions

This study provides the first detailed description of the regional and cellular expression of DAO and SRR in the human brain. The findings suggest that d-serine synthesis is primarily glial and occurs in the cerebellum as well as the cerebral cortex. DAO expression and thence d-serine catabolism is also glial in the cerebellum, and given the cellular expression of SRR, may regulate d-serine levels at localized sites such as the Bergmann glia. However, in the cortex DAO is neuronal, suggesting a different catabolic route, although the lack of cortical DAO activity, despite robust expression, remains a paradox.

This study demonstrated increased expression in schizophrenia of SRR in the DPFC, and of DAO in the cerebellum. The results provide some support for theories of aberrant d-serine metabolism and NMDAR dysfunction in the disorder (Olney & Farber, 1995; Coyle, 1996; Tsai *et al*., 1998; Hashimoto *et al*., 2003, 2005; Moghaddam, 2003; Heresco-Levy *et al*., 2005). However any pathophysiological interpretation remains speculative given the various uncertainties and inconsistencies dicussed here.

## Abbreviations

DAO: d-amino acid oxidase
dg: dentate gyrus
DPFC: dorsolateral prefrontal cortex
GAPDH: glyceraldehyde-3-phosphate dehydrogenase
gcl: granule cell layer
gm: grey matter
ml: molecular layer
NMDAR: *N*-methyl-d-aspartate receptor
PBS: phosphate-buffered saline
PBS-T: PBS−0.3% Triton X-100
PCR: polymerase chain reaction
pcl: Purkinje cell layer
r18s: ribosomal 18s
rp: Bergmann glia radial processes
SDS: sodium dodecyl sulphate
SRR: serine racemase
SSC: standard saline citrate
wm: white matter.
